# The Will of Sir Erasmus Wilson

**Published:** 1885-01

**Authors:** 


### SELECTIONS.

---

THE WILL OF SIR ERASMUS WILSON.

The will, with a codicil, of Sir James Erasmus Wilson, F. R. S., F. R. C. S., late of No. 17 Henrietta street, Cavendish square, who died on August 8th last, at Westgate-on-Sea, was proved on the 17th ultimo by Henry Palfrey Stephenson, Frederick Lane Linging, and Charles Alfred Swineburne, the executors, the value of the personal estate amounting to upwards of £264,000. The testator bequeaths three pictures: A sea-piece, by Salvator Rosa; "Thiee Sheep," by Sidney Cooper; and a "Hurricane in the Bay of Biscay," by E. W. Cook, to his wife, Dame Charlotte Mary Wilson, for life, and then to the South Kensington museum, or such other public institution in Great Britain as his trustees may think most desirable in the interests of the public—£500. The remainder of his pictures, and all his furniture, plate, works of art, horses and carriages, to his wife; an annuity of £500 to his brother, and legacies to his executors. All his real and household estate, and any personal estate savoring of realty, he gives to his wife, absolutely. The residue of his personal estate he leaves, upon trust, to pay the income to his wife, for life; and, at her death, £5,000 each, free of legacy duty, to the Royal National Hospital, or Sea-Bathing Infirmary, for scrofula only, at Margate; the Royal Medical Benevolent College, the Medical Benevolent Fund, and the Society for the Relief of the

Widows and Children of Medical Men; and the whole of the ultimate residue to the Royal College of Surgeons.

*Gifts to Medical Colleges.*—The brilliant example of Sir Erasmus Wilson has been nobly imitated across the Atlantic. Mr. W. H. Vanderbilt has made a gift of half a million dollars to the College of Physicians and Surgeons, New York, an institution seventy-seven years old, whose career during more than three-quarters of a century has been an almost constant endeavor to equip its pupils with the best medical education that the condition of the United States would allow. Great as will be the immediate beneficial results of this magnificent gift, Dr. Austin Flint observes in the *New York Medical Journal:* "We may hope for still greater benefit from it as tending to give to private beneficence a direction towards the promotion of medical knowledge and medical education." Mr. Carnegie, of New York, has likewise set a good example in the same direction, by a munificent donation to the Bellevue Hospital Medical College. Our American contemporary, above quoted, makes the following pertinent observations on these acts of bounty to the medical profession: "We are not of those who ignore the evils that are thought to go hand in hand with the amassing of huge fortunes by individuals—we believe them to be real and not imaginary; but it is a great mitigation of those evils for a man of vast wealth to give a tithe of his possessions to the cause of suffering humanity. It is one of those touches of nature that make the whole world kin. The humblest beneficiary of medical care—and they are to be counted every year by the million—may now profit directly, in the heightened quality of his dole, by this one act of a rich man. Surely such a deed should go far to reconcile the poor to a splendor they must look upon but may not share. Let us hope that, so far as material benefactions are concerned, medicine has at last entered upon an era of good fortune; and, indeed there seems to be good ground for the hope when, within the space of a single year, Sir Erasmus Wilson's munificent bequest to the Royal College of Surgeons, Mr. Carnegie's gift to theBellevue Hospital Medical College, and now this princely endowment of the College of Physicians and Surgeons by Mr. Vanderbilt, have all been chronicled." There must be numbers of rich people who make wills and leave money to those about whom they care little, or to further objects of far less importance to humanity than the advancement of the science and art which benefits all mankind. Not forgetting the instance of the Lariboisière Hospital, and several other continental medical charities, it is chiefly amidst Anglo-Saxon communities that the rich and bountiful have done so much good in supporting and endowing institutions for the cure of the sick. It is also in the two great Anglo-Saxon nations, and simultaneously, that a brilliant innovation has been introduced, the endowment of learned bodies that qualify citizens for the profession of healing their countrymen. It is sincerely to be desired that the example of Wilson, Vanderbilt, and Carnegie will be freely followed in the British empire and in the United States.—*From the British Medical Journal.*

